# The rise of diagnostic identities in society

**DOI:** 10.1371/journal.pmen.0000779

**Published:** 2026-11-03

**Authors:** Lars Veldmeijer, Jim van Os

**Affiliations:** 1 Research Group Care and Innovation in Psychiatry, NHL Stenden University of Applied Sciences, Leeuwarden, The Netherlands; 2 Division Neuroscience, UMC Utrecht, Utrecht, The Netherlands; PLOS: Public Library of Science, UNITED KINGDOM OF GREAT BRITAIN AND NORTHERN IRELAND

Ten years ago, Brinkmann [1] described how psychiatric concepts were being used to interpret everyday experiences and forms of suffering. Today, this development appears to have gained further momentum, as people increasingly adopt psychiatric narratives to explain everyday, non-psychiatric problems. As a sign of our times, The New Yorker published in 2024 the article *‘Why We’re Turning Psychiatric Labels Into Identities’* [2]. While this is partly due to how systems are organized – a person must first have a diagnosis to be eligible for covered care, and to receive a diagnosis, the problems a person has must be translated into diagnostic language – this process is accelerated by digital culture, where self-diagnosis and identity-based diagnostic frameworks are widely promoted [3]. The diagnostic system, however, is not designed to shape and validate people’s identities [4]. As lived experience perspectives suggest [5], overidentifying with diagnostic concepts can lead to a so-called ‘diagnostic identity’ [6] that, when fully internalized, can become difficult to shake off [5]. While people’s struggles and challenges are undoubtedly real and should not be disregarded, we are concerned that people increasingly turn to diagnostic concepts for relief and come to identify with them.

In the case of mental health problems, there seems to be a powerful mechanism at work wherein people's pre-diagnostic self-conception differs from their post-diagnostic one (sometimes for the better and sometimes for the worse; e.g.[7],). We have sought to explain the negative impact of this mechanism by introducing the term ‘identity intervention,’ that is, an intervention that influences the self-concept, expectations, and life story of a person with mental health issues in a potentially harmful way that goes beyond the descriptive function of the diagnostic label (also applicable to self-diagnosis) [8]. Our concept is similar to Hacking’s theory of “dynamic nominalism” [9]. According to his philosophy, classifications influence how people (causally) understand themselves and change the kind of people they are, while these changes, in turn, can alter the meaning and application of these classifications, resulting in “looping effects” [9].

Correspondingly, scholars have argued that internalizing classifications can be world-disclosing and shape identity and life expectations in restrictive ways [10], or worse, ‘cage’ – metaphorically speaking – recipients in diagnostic concepts [11]. Consider, for example, someone who, after a period of intense struggling and impairment, is diagnosed with a mental health condition. The classification may offer them immediate relief and a socially accepted language to describe the experiences that led to a decline in their quality of life (see [12] for the potential therapeutic benefits). Over time, however, the diagnostic lens may extend to an increasing range of everyday experiences. If the concept is perceived as explanatory rather than descriptive, it can also become part of one’s sense of self [5], as if it captures one’s very essence. For people who have internalized a diagnostic classification as part of who they are, being told that these classifications merely describe patterns and do not explain an underlying condition can feel like a denial of their explanatory model and, by extension, their identity.

A broader problem with current diagnostic language is therefore that it obscures the difference between describing and explaining for the general public, but also for mental health professionals. The recently published *Independent review into mental health conditions, ADHD and autism* [13], also known as the Fonagy review, is a timely example. Although the report’s working definition of diagnosis is pragmatic and refers to internationally recognized criteria for diagnostic categorization (p. 49), its earlier statement that diagnosis “[…] *explains* the nature of a condition (italics by us) (p. 32)” sits uneasily with this definition, blurring the distinction between *describing* patterns of experiences and behaviours and *explaining* the nature of such patterns. The Fonagy review illustrates how diagnostic concepts can easily become quicksand, making clear communication difficult, even for the most distinguished mental health experts. It is thus understandable that the general public struggles to discern what these concepts can and cannot do.

In light of these insights, is it too far-fetched to hypothesize that the dominance and ambiguity of diagnostic language in society, alongside the broadening of diagnostic concepts [6], may contribute to worsening mental health? We don’t think so. Both from the bottom-up and the top-down, people are influenced and incentivized to relate to and behave in accordance with diagnostic concepts [8]. People rethink themselves in response [9]. Moreover, society has – through the process of psychiatrization – adopted, reified, incorporated, and commodified diagnostic concepts across diverse domains of public life, becoming increasingly attached to them (e.g., social media, everyday conversations, popular culture and journalism, education, the workplace, identity-based communities and advocacy, self-help and the wellbeing industry, and public policy, law and social services) [14]. Hence, we observe a self-reinforcing loop in which both society and the mental health care system struggle to view and communicate experiences of mental health difficulties separately from diagnostic concepts, sometimes treating these concepts as definitive explanations, leaving “patients and the public […] with a picture of the mind that is far too simple” [15].

If we want to break this loop, prevent a potential ‘epidemic’ of diagnostic identities, and remain attentive to the broader historical and social forces that influence our lives [1], perhaps the first step is to create space for other ways of articulating mental distress and suffering in daily life, without feeling the need to resort to clinical terminology.

Are we, as a society, however, still capable of talking about and making sense of human suffering without relying on diagnostic concepts?
